# Risk Factors for Child Mortality in the Kassena-Nankana District of Northern Ghana: A Cross-Sectional Study Using Population-Based Data

**DOI:** 10.1155/2018/7692379

**Published:** 2018-08-01

**Authors:** Michael N. K. Babayara, Bright Addo

**Affiliations:** ^1^Postgraduate College, 37 Military Hospital, Accra, Ghana; ^2^University of Ghana School of Public Health, Accra, Ghana

## Abstract

Child mortality continues to be a major public health problem in Ghana, especially in northern Ghana where child survival rates are among the lowest. Though strategies are in place to address it, progress made is unsatisfactory and the Sustainable Development Goal 3 risks being missed. This makes the reexamination of the risk factors for child mortality crucial as results will aid in the modification of existing strategies aimed at addressing the problem. This study was a population-based case control study utilizing data (2007–2011) from the Demographic Surveillance System database of the Navrongo Health Research Center. Cases and controls were selected from the database and analysed unmatched. Cases were children who died before age five and controls were live children within the same year group. Univariate and bivariate analyses were performed using STATA (v13). The results revealed the main causes of death in the area to include malaria, diarrhoeal diseases, respiratory infections, and malnutrition. Mother's age at birth, mother's educational level, and mother's household socioeconomic status were significantly related to child mortality. On the basis of these results, we conclude that the known risk factors for child mortality in the Kassena-Nankana district have not changed much over the years. Current child survival strategies therefore need to be evaluated and modified where necessary to yield desired results.

## 1. Introduction

The death of children before their 5th birthday still remains a major public health priority [1]. Apart from the fact that children under five contribute to an estimation of life expectancy at birth, they are also a key health indicator in any country. A 2013 UNICEF report estimated that 6.6 million children die per year, 546,000 per month; 125,000 per week; 18000 per day; and 12 per minute [2]. Put in more practical terms, a child dies each time one takes a breath.

Currently, it is estimated that the total number of under-five deaths dropped from 12.6 million (35,000 every day) in 1990 to 5.6 million (15,000 every day) in 2016 [3].

More than half of these deaths are preventable, and the description of the situation as a public health disaster by some researchers in the early 2000s [4] is very appropriate even today. Globally, the sub-Saharan Africa region continues to remain the region with the highest child mortality rates. In 2016, the region recorded an estimated average mortality rate of 79 per 1,000 live births. This figure translates into 1 in 13 children dying before their 5th birthday. It is 15 times higher than the average of 1 in 189 in high-income countries and 20 times higher than New Zealand and Australia [3].

This notwithstanding, the UNICEF suggests that progress is being made in all countries towards addressing the problem since the global average has dropped from 93 per 1000 live births in 1990 to 41 per 1000 in 2016, a 56.0% decline [3]. Sadly though, at the current rate of improvement, the Sustainable Development Goal on child mortality is likely to be missed [3].

Studies to investigate and understand the risk factors for infant and child mortality abound with factors such as maternal age at the birth of the child [5, 6], maternal education [1], parity [7, 8], family size [9], birth interval [9, 10], access to health care [11], and factors surrounding the prenatal and postnatal periods being implicated to varying degrees. Though these risk factors have been implicated in various studies, it is worth noting that their distribution in time, place, and person varies from locality to locality. Addressing child mortality at any particular place therefore requires an understanding of the particular risk factors prevalent there.

In Ghana, child mortality has always been an issue of public health interest. Though there has been a general downward trend in the mortality figures since the late 1980s, the rate of decline has not been satisfactory. Between 1988 and 2003, the rate of decline significantly slowed down, and the current rate of about 74 deaths per 1000 live births [2] still leaves much to be desired. The northern parts of the country where poverty and general standards of living are comparatively worse have been reported to register some of the highest mortality rates in Africa [12].

Apart from diarrhoeal diseases and infections, malnutrition was found to be a major risk factor in Ghana [13]. The rollout of the Ghana vitamin A supplementation Trial (Ghana VAST) in Navrongo in the early 1990s was therefore crucial. It led to a 19.0% reduction in child mortality in the study area and formed the basis for subsequent provision of vitamin A supplements to children across the country. This provides proof that with an understanding of the main risk factors, public health interventions can be well targeted to bring change.

It was on this basis that this study was designed; a follow-up study to an earlier one conducted in 1995 by Binka and colleagues in which a variety of risk factors were investigated in the Kassena-Nankana District (KND) of the Upper East region of Ghana using a matched case control study [14].

Binka and colleagues found positive associations with mortality in cases where deliveries were conducted by untrained persons, the preceding birth interval was less than 24 months, the mother suffered domestic violence from the father, and the source of water was unprotected. However, they found no association linking child mortality to weaning practices, parental education, or any of the socioeconomic or hygiene variables they considered [14]. The study concluded and recommended targeted interventions to include increased supervision of deliveries by trained people and a general improvement in socioeconomic conditions.

Almost two decades following that study, so much is reported to have changed in the socioeconomic, cultural, and religious circumstances of the people of the KND. For instance, there is now a more improved primary health-care system following the successful country-wide rollout of the CHPS compound initiative [15]. Fertility rates in the Kassena-Nankana District have decreased while contraceptive use has increased [16]. People have been observed to be inclined towards Christianity and Islam while deserting the hitherto widely practiced traditional African religion [17]. Maternal mortality declined by 40.0% when the figure for years 2002–2004 is compared to that for years 1995-1996 [18]. The practice of infanticide, otherwise known as the “spirit child phenomenon,” reduced from 15.0% in the 1990s [19] to 4.9% in 2002 [20]. Similar improvements have been reported in many other variables that are bound to have an impact on the overall health status of the people.

Given the transformations in the socioeconomic, cultural, and demographic characteristics of the area, risk factors identified by Binka et al. may have also changed over the period. We therefore through this study assessed the current potential risk factors contributing to child mortality in the Kassena-Nankana district of Northern Ghana.

## 2. Materials and Methods

### 2.1. Study Area

The Kassena-Nankana district is primarily a rural district with a total population of 152,000, an estimated 10.0% of which live in the district capital, Navrongo [21]. The district is in the extreme north eastern corner of Ghana and lies within the Guinea savannah woodland area of the country, covering an area of 1672 square km.

The area lies in the meningitis belt and so experiences perennial outbreaks of cerebrospinal meningitis, sometimes with devastating fatalities. The settlement pattern in the district is mostly dispersed with extended family units living together in compound houses surrounded by farmlands. The main occupation is subsistence farming, with cereals and vegetables forming the majority of crops cultivated. Livestock rearing is also widely practiced.

Though generally deprived, the district is endowed with a number of health and educational institutions. Notable health institutions are the War Memorial hospital (the district hospital) and the renowned Navrongo Health Research Center (NHRC). There are mission clinics, private clinics, several health centres, and CHPS compounds distributed across the district.

Educational institutions include several senior high schools, a teacher training college, a community health nurses training college, and a university campus.

The Navrongo Demographic Surveillance System (NDSS) run by the Navrongo Health Research Centre routinely captures and updates data on sociodemographic and health indices of the district on a quarterly basis [22].

### 2.2. Study Population

Babies and children aged between 1 month and five years, both dead and alive in the study area, constituted the study population. The sampled population was babies and children both dead and alive in this age category from January 2007 to December 2011.

In the study area, a system exists where field staff from the Demographic and Health Surveillance System of the Navrongo Health Research Centre visit each household every four months to collect data on vital events and various health and sociodemographic variables of individuals in the district. Where deaths are reported, field staff are sent to interview the guardians and relatives of the deceased within two months of the death, to ascertain the circumstances leading to death and the symptoms and signs associated with the death using verbal postmortem forms. These forms get coded independently by three medical doctors in an attempt to assign a most probable cause of death to each using a standard disease classification system (The International Classification of Diseases). With these coded verbal postmortems, most likely causes of death can be found and assigned to each death. These are maintained in the Demographic Surveillance System (DSS) database.

### 2.3. Sampling

All deaths that were captured and recorded in the DSS database for children aged between one month and fifty-nine months in the study area for the period 1st January 2007 to 31st December 2011 were used for this analysis. Cases were deaths between 1 and 59 months, and controls were live children in the same cohort.

For eligibility to be sampled, a case should have died in the postneonatal period and should have been resident in the study area for at least 6 months before death. Controls were all the children in the stated age group within the study period. In the study area, neonatal deaths are often underreported because of a belief that children who die in the perinatal/neonatal period “did not come to stay” and so are not considered as children. Also, where children were killed in the practice of infanticide, their deaths are usually not reported. To avoid skewing the results due to underreporting of neonatal deaths, we excluded neonates from our analysis.

### 2.4. Variables

The dependent variable was death of child which was categorized and coded as “0 = Not dead” or “1 = Dead”. The independent variables (risk factors) extracted for the relational tests included sex of child (coded as “M = Male, F = Female”), mother's age at birth (coded as “1 = 0–19, 2 = 20–29, 3 = 30–39, 4 = 40–49, 5 = 50+), mother's educational level (coded as “1 = None, 2 = Primary, 3 = Junior High School, 4 = Senior High School/Tertiary), residence (coded as “1 = Urban, 2 = Rural), and mother's household socioeconomic status (SES) (coded as “1 = Least poor, 2 = Less poor, 3 = Poor, 4 = More poor, 5 = Very poor).

### 2.5. Data Collection and Statistical Analysis

Data on potential risk factors were extracted from the DSS database for all cases and controls. Data extracted mainly covered sociodemographic variables of the children and their mothers.

Results of verbal postmortems where available were also generated from the database for all cases and used for analysis. The Navrongo Health Research Centre collects data on a quarterly basis on mortalities within the district. Using the WHO tool for verbal autopsies, possible causes of death are assigned to individuals who do not have a death certificate issued by a doctor. STATA version 13 was used for analysis. The data extracted from the DSS database were imported into the software and cleaned. Sociodemographic variables were analysed descriptively, and results were reported in frequencies and percentages. Relational tests between the independent variables (risk factors) and dependent variable (death of child) was performed using the chi-square test of independence (*χ*^2^). All relational tests were conducted at the 95% significance level.

### 2.6. Ethical Issues

The Navrongo Health Research Centre sought consent from the Ministry of Health at the time of the establishment of the DSS in 1993. Then, the chiefs and people of the Upper East region consented verbally at durbars, and compound and household heads consented verbally at the time of every interview [23]. Ethical clearance for data collection was granted by the ethics review committee of the research centre. Permission was sought from the Director of the Navrongo Health Research Centre before data were extracted and used for this study.

## 3. Results

### 3.1. Sociodemographic Characteristics

A total of 15745 children aged between 1 month and 5 years were captured in the DSS database between January 1997 and December 2011, and 967 (6.14%) of them died before age five, giving a child mortality rate of 61.4 per 1000 live births over the period. More males died (53.3%) than females (46.7%). The ages at death of these children ranged from 29 days to 1756 days with a median age of 466 days. Majority of the deaths occurred among the 1–4 years age cohort. Majority (89.5%) of the deaths were reported from rural areas, and most (55.2%) of these deaths occurred at home. The distribution of deaths varied according to seasons, with majority (60.3%) occurring during the wet season (Table 1).

Maternal characteristics such as education, age at birth of the child, and household socioeconomic status (SES) were considered as these have been implicated in other studies. Mothers in the 20–29 years age group had the highest frequency of dead children (44.7%). Mothers whose children died the most had no formal education (82.2%). Comparatively, a high percentage (29.0) of children who died were born to mothers who belonged to the least poor SES category (Table 1).

### 3.2. Causes of Death

Malaria, diarrhoeal diseases, respiratory infections, anaemia, and malnutrition were the top 5 leading causes of child mortality in the district. Malaria alone accounted for 38.2% of the deaths followed by intestinal infectious diseases (dominant being diarrhoeal diseases) which constituted 11.1%. Acute respiratory infections, anaemia, and malnutrition accounted for 8.3%, 4.2%, and 2.8%, respectively. The other five causes were other infections, meningitis, the spirit child phenomenon, accidental drowning, and submersion (Table 2).

### 3.3. Risk Factors Associated with Child Mortality

Results of the bivariate analysis conducted using the chi-square test of independence revealed mother's age at birth, mother's educational level, and mother's household SES to be significantly related to child mortality (Table 3).

## 4. Discussion

It has been 16 years since world leaders committed to the Millennium Development Goal 4 (MDG), which set out to reduce the under-five mortality rate by two-thirds between 1990 and 2015, and 2 years since a similar commitment was made towards the Sustainable Development Goal (SDG) target on child survival, which is to reduce neonatal mortality to at least 12 deaths per 1000 live births and under-five mortality to at least 25 deaths per 1000 live births. Two years after the commitment to the SDG target, present statistics indicate that the world has made substantial progress in reducing child mortality, with the total number of under-five deaths dropping to 5.6 million in 2016 from 12.6 million in 1990 [3]. Despite this progress, large disparities still exist in the survival chances of children aged 1–4 across regions and countries. In sub-Saharan Africa, approximately 1 child in 13 dies before his or her firth birthday, while in the world's developed/high-income countries, the ratio is 1 in 189. Among neonates, the gap is even wider as in sub-Saharan Africa, about 1 child in 36 dies in the first month, while in the developed/high-income countries, the ratio is 1 in 333 [3]. In Ghana, considerable progress has been made towards reducing infant and child mortality rate in the last three decades. For example, the infant mortality rate (IMR) was close to 100 deaths per 1000 live births in the late 1970s, and towards the end of the 1990s, the IMR declined to 70 deaths per 1000 live births and further to a 50 per 1000 live births in 2008 [24]. Disparities have also been observed in the regional distribution of infant and child mortality rates in Ghana. Both IMRs and U5MRs have been reported to be consistently the highest in the Upper West and Upper East regions [25]. With the introduction of interventions such as the Vitamin A Supplementation Trail (1989–1992), which led to a 20% reduction in under-five mortality, the Insecticide-treated Bednet Trial (1993–1995), which also led to a 17% reduction in child mortality and the Navrongo Community Health and Family Planning Project (1994–2003), which resulted in a 40% reduction in child mortality in the KND [26], one would have thought that rate of child mortality in the district would have drastically reduced by now. However, this is not the case as the 61.4 per 1000 live births rate we found in this study is still high despite an improvement on the 2003 rate which was 79 per 1000 live births. Compared to the national rate of 59 per 1000 live births [3], the area lags that of the whole country in the quest towards achieving the SDG on child survival. The implication of this result is that, an evaluation of these infant and child mortality reducing strategies in the KND is required. This evaluation would help identify challenges associated with the continuous implementation of these strategies and possible solutions to resolving these challenges.

Acting singly, or together, we identified the main causes of child mortality in the KND to include malaria, diarrhoea, pneumonia, and anaemia. In the sub-Saharan Africa region, pneumonia, diarrhoea, and malaria remain among the leading causes of death among children under age 5, with these causes accounting for almost a third of the global under-five deaths and about 40.0% of under-five deaths in the region [3]. Accelerating the reduction in child mortality in the Kassena-Nankana district would therefore require effective preventive and curative interventions that target these main causes of child deaths. Though still among the top 10 killers of children in the district, it is worth mentioning that the spirit child phenomenon which was found to contribute to about 1.7% of deaths in this study is a decline from the 15.0% found by Allotey and Reidpath in 2001 [19], which is encouraging and shows that progress is being made albeit slow at stamping out the practice from the culture of the people.

Risk factor studies on child mortality have been equivocal in reporting factors such as sex of child, age of mother at birth of child, educational attainment of mother, household socioeconomic status, and residence to be significantly related to child mortality. Consistent with these findings, our study also found a significant relationship between mother's age at birth of child, mother's education level, and maternal household socioeconomic status to be significantly related to child mortality in the KND. We found factors such as sex of child and residential dwelling, however, not to be significantly related to child mortality. Demographic data have over the years predicted better survival chances for female children than their male counterparts. Though we found a nonsignificant relationship between sex and child mortality, a higher percentage of male children died than their female counterparts. While empirical explanations to this gender difference in child mortality is still lacking, many believe that the boy child being more adventurous than the girl child is more likely to be involved in trauma situations, pick up infections, and so on.

The best age at which to give birth for a woman is generally considered to be from midtwenties to midthirties. Most women within this age group would have finished schooling or professional training and would have been ready to get married. Also due to biological maturation, women within this age bracket would have been physically and psychologically ready for the task of child care. In our study, women within the age group of 20–29 years and those between 30 and 39 years had less of their children dying. While the percentage of children who died was 5.9% for mothers between 20 and 29 years, that of mothers between 30 and 39 years was 5.7%. Child mortality in our study was the highest for mothers in their teen years and those in their late 40s. The relative ignorance of a young mother and the increased risk of congenital defects in the unborn babies to mothers conceiving after age 35 years could have accounted for this high figure. Additionally, advancing age in general is associated with medical conditions such as diabetes mellitus and hypertension which affect the health of the unborn baby in an affected pregnant woman. This may therefore contribute to child deaths especially during their earlier days in life.

Maternal education has been shown by various studies to contribute significantly to a child's chances of survival. The conclusion reached by many researchers which has been corroborated by results of this study indicates that when a woman has had some level of formal education, her children have a better chance at surviving. In this study, we found child mortality to be highest among mothers with no formal education, and the percentage of death decreased with an increase in educational level of a mother. Education does not only empower the female economically, but also enables the mother to make reasonable decisions regarding her family and children to enhance their survival. It is therefore not surprising that many schools of thought have continued to call for efforts to be doubled to encourage the education of the girl child.

Residence in this study did not show a significant relationship with child mortality as other studies have. In spite of this nonsignificance, we found the percentage of child mortality to be higher among mothers who were rural dwellers. This finding is not surprising as the Kassena-Nankana District is generally a homogenous society with regards to socioeconomic development. The differential benefits that would have inured to the benefit of urban dwellers such as better health facilities, better education, and so on are therefore almost evenly distributed such that the same effect is seen on mortality indices. Rural dwelling has been associated with higher levels of poverty and lack of basic social amenities coupled with unhealthy cultural practices which increases the risk of morbidity and mortalities across various age strata.

The effect of poverty on child mortality can be seen to cut across national, regional, district, and family boundaries. At the international level, developed and richer countries generally have lower mortality rates than poorer countries. At the national level, richer communities have lower rates than poorer ones, and at the community level, richer families tend to have better health and survival rates than poorer ones. We found household socioeconomic status of mothers to be significantly related to child mortality. The richer a household is, the better the health care, nutrition, and education which all directly impact mortality. It is therefore in the partial interest of child survival that there is a Sustainable Development Goal targeting poverty. Realizing this goal will go a long way to improve child survival.

### 4.1. Limitations and Strengths

Like any other empirical study, our study has limitations, and it is important to mention its key ones. First, the use of a preexisting dataset did not permit us to examine the influence of other risk factors beyond what was captured in the dataset. Second, the aggregation of the study sample in one group, that is, “child not dead” did not permit a multivariate analysis (binary logistic regression) to further identify significant determinants of child mortality. The limited data on neonatal deaths prevented us from including this important group in our analysis.

Lastly, the study results relate specifically to the Kassena-Nankana district, and this limits generalization to the entire Ghanaian population.

Despite these limitations, our study is the first after the 1995 study of Binka and colleagues to reexamine the risk factors for child mortality in the KND considering the changes that have occurred in the social, economic, and cultural landscape of the district. Findings of our study are therefore important and will contribute significantly to policy reforms.

## 5. Conclusion

The struggle to improve child survival in Ghana has been a long one and though slow, progress is being made across all regions in the country. In this study, we found the child mortality rate in the KND to be 61.4 per 1000 live births which is an improvement on the 79 per 1000 live births in 2003 [23]. This figure is also lower than the national rate for the period 2009 to 2011 (UNICEF, 2011). Though the reason for this progress is not immediately known, it is important that efforts are made to sustain the progress. Considering the fact that malaria was identified to be the leading cause of child mortality in the area, it is recommended that existing strategies to curb malaria in the district such as indoor residual spraying, distribution of insecticide-treated bed nets, and Intermittent Preventive Treatment during Pregnancy (IPTp) be evaluated and strengthened. Efforts on improving vaccine coverage, especially for rotaviral, pneumococcal, and meningococcal vaccines should be scaled up as intestinal infectious diseases (including diarrhoeal diseases), acute respiratory infections (including pneumonia), and anaemia were found to be other leading causes of child mortality in the district.

For the Kassena-Nankana district, many socioeconomic transformations that have reportedly taken place in the area have not changed these factors to a large extent. We found the educational attainment of mothers in the district to be very low, especially at the Junior High, Senior High, and tertiary levels, and since maternal education was found to be significantly related to child mortality, we recommend that advocacy on girl child education in the district be improved. Increased girl child enrolment at the secondary and tertiary levels should be the focus of such advocacy because education up to these levels will affect child mortality in two ways: on one hand, it has a direct potential for reducing child mortality as shown in our study, and on the other hand, it increases the maternal age at birth of child which was also found to be significantly related to child mortality in our study.

Lastly, as malnutrition was identified as the firth cause of child mortality in the district, we recommend that livelihood empowerment programmes aimed at improving the socioeconomic status of women in the district should be evaluated and improved upon. When mothers are economically empowered, they are able to feed themselves and their children.

## Figures and Tables

**Table 1:** Descriptive data on background characteristics of children and mothers (*N*=15745).

Characteristics	Children dead (*n*=967)	Children not dead (*n*=14778)
	*n* (%)	*n* (%)
*Children*		
Sex		
Female	452 (46.7)	7272 (49.2)
Male	515 (53.3)	7506 (50.8)
Age group		
1–11 months	391 (40.4)	2980 (20.2)
1–4 yrs	576 (59.6)	11789 (79.8)
Residence		
Urban	102 (10.5)	1842 (12.5)
Rural	865 (89.5)	12936 (87.5)
Place of death^a^		
Hospital	277 (34.5)	
Health centre	44 (5.5)	
Home	444 (55.2)	
Traditional birth attendant	2 (0.2)	
Other	37 (4.6)	
Season of death of child^a^		
Wet	583 (60.3)	
Dry	384 (39.7)	
*Mothers*		
Age of mother when child was born		
0–19	121 (13.1)	1368 (9.9)
20–29	412 (44.7)	6526 (47.4)
30–39	267 (29.0)	4382 (31.9)
40–49	109 (11.8)	1349 (9.8)
50+	12 (1.3)	131 (1.0)
Age of mother when child died^b^		
0–19	61 (6.6)	
20–29	403 (43.8)	
30–39	293 (31.8)	
40–49	146 (15.9)	
50+	18 (2.0)	
Educational level of mother		
None	757 (82.2)	10400 (75.6)
Primary	110 (11.9)	2192 (15.9)
Junior high school	37 (4.0)	811 (5.9)
Senior high school/Tertiary	17 (1.8)	352 (2.6)
Mother's SES		
Least poor	274 (29.0)	3811 (26.5)
Less poor	240 (25.4)	3189 (22.2)
Poor	225 (23.8)	3109 (21.6)
More poor	131 (13.9)	2726 (18.9)
Very poor	75 (7.9)	1557 (10.8)

^a^Data exclude live children. ^b^Data exclude mothers with live children.

**Table 2:** Causes of child mortality in the Kassena-Nankana district from January 2007 to December 2011.

Causes	Frequency (*n*=967)	Percent
Malaria	369	38.2
Intestinal infectious diseases (incl. diarrhoeal diseases)	107	11.1
Acute respiratory infections (incl. pneumonia)	80	8.3
Anaemia	41	4.2
Malnutrition	27	2.8
All other specified infectious and parasitic diseases	23	2.4
All other unspecified infectious and parasitic diseases	19	2.0
Spirit child	16	1.7
Meningitis	13	1.3
Accidental drowning & submersion	12	1.2
Other unintentional causes	12	1.2
Other disorders of the nervous system	10	1.0
Acute abdominal conditions (incl. intestinal obstruction)	8	0.8
All other specified causes	8	0.8
Unspecified noncommunicable disease	7	0.7
All congenital malformations	6	0.6
Insufficient information/specific information missing	6	0.6
Disorders of kidney (excl. obstructive disorders)	5	0.5
Accidental poisoning & exposure to noxious substance	4	0.4
All other gastrointestinal disorders	4	0.4
No information (includes refusals, no informant found)	4	0.4
Other disorders related to perinatal period	3	0.3
Other respiratory system illness (excluding ARI, tuberculosis, and neoplasms)	3	0.3
Transport accident	3	0.3
Accidental exposure to smoke, fire, and flames	2	0.2
Accidental fall	2	0.2
All other malignant neoplasm	2	0.2
Epilepsy	2	0.2
HIV/AIDS	2	0.2
Rabies	2	0.2
Neonatal sepsis	1	0.1
Prematurity and low birth weight	1	0.1
Nonassigned causes (no verbal PM or cause of death not found)	163	16.9

**Table 3:** Bivariate analysis of risk factors and child mortality (*N*=15745).

Characteristics	Children dead (*n*=967)	Children not dead (*n*=14778)	*p* value for *χ*^2^ test
	*n* (%)	*n* (%)	
Sex of child			
Female	452 (5.9)	7272 (94.1)	0.137
Male	515 (6.4)	7506 (93.6)	
Mother's age at birth			
0–19	121 (8.1)	1368 (91.9)	0.002^*∗*^
20–29	412 (5.9)	6526 (94.1)	
30–39	267 (5.7)	4382 (94.3)	
40–49	109 (7.5)	1349 (92.5)	
50+	12 (8.4)	131 (91.6)	
Mother's educational level			
None	757 (6.8)	10400 (93.2)	0.000^*∗*^
Primary	110 (4.8)	2192 (95.2)	
Junior high school	37 (4.4)	811 (95.6)	
Senior high school/Tertiary	17 (4.6)	352 (95.4)	
Residence			
Urban	102 (5.3)	1842 (94.7)	0.079
Rural	865 (6.3)	12936 (93.7)	
Mother's SES			
Least poor	274 (6.7)	3811 (93.3)	0.000^*∗*^
Less poor	240 (7.0)	3189 (93.0)	
Poor	225 (6.8)	3109 (93.2)	
More poor	131 (4.6)	2726 (95.4)	
Very poor	75 (4.6)	1557 (95.4)	

^*∗*^ *p* < 0.05.

## Data Availability

The data used to support the findings of this study are available from the corresponding author upon request.
